# [1,2,4] Triazolo [3,4-*a*]isoquinoline chalcone derivative exhibits anticancer activity via induction of oxidative stress, DNA damage, and apoptosis in Ehrlich solid carcinoma-bearing mice

**DOI:** 10.1007/s00210-022-02269-5

**Published:** 2022-07-26

**Authors:** Amr Ahmed WalyEldeen, Haidan M. El-Shorbagy, Hamdi M. Hassaneen, Ismail A. Abdelhamid, Salwa Sabet, Sherif Abdelaziz Ibrahim

**Affiliations:** 1 Zoology Department, Faculty of Science, Cairo University, Giza, 12613 Egypt; 2 Faculty of Biotechnology, October University for Modern Science and Art (MSA), 6th October, Cairo, Egypt; 3 Department of Chemistry, Faculty of Science, Cairo University, Giza, 12613 Egypt

**Keywords:** Chalcones, Chemotherapy, Ehrlich solid carcinoma, DNA damage, Oxidative stress, Apoptosis

## Abstract

Despite the advances made in cancer therapeutics, their adverse effects remain a major concern, putting safer therapeutic options in high demand. Since chalcones, a group of flavonoids and isoflavonoids, act as promising anticancer agents, we aimed to evaluate the in vivo anticancer activity of a synthetic isoquinoline chalcone (CHE) in a mice model with Ehrlich solid carcinoma. Our in vivo pilot experiments revealed that the maximum tolerated body weight-adjusted CHE dose was 428 mg/kg. Female BALB/c mice were inoculated with Ehrlich ascites carcinoma cells and randomly assigned to three different CHE doses administered intraperitoneally (IP; 107, 214, and 321 mg/kg) twice a week for two consecutive weeks. A group injected with doxorubicin (DOX; 4 mg/kg IP) was used as a positive control. We found that in CHE-treated groups: (1) tumor weight was significantly decreased; (2) the total antioxidant concentration was substantially depleted in tumor tissues, resulting in elevated oxidative stress and DNA damage evidenced through DNA fragmentation and comet assays; (3) pro-apoptotic genes *p53* and *Bax*, assessed via qPCR, were significantly upregulated. Interestingly, CHE treatment reduced immunohistochemical staining of the proliferative marker ki67, whereas BAX was increased. Notably, histopathological examination indicated that unlike DOX, CHE treatment had minimal toxicity on the liver and kidney. In conclusion, CHE exerts antitumor activity via induction of oxidative stress and DNA damage that lead to apoptosis, making CHE a promising candidate for solid tumor therapy.

**Supplementary Information:**

The online version contains supplementary material available at 10.1007/s00210-022-02269-5.

## Introduction

Cancer is a devastating disease, and, globally the second leading cause of death (Siegel et al. 2019). In 2020, 19.3 million patients were newly diagnosed with cancer and approximately 10 million deaths were cancer related (Sung et al. 2021). For several decades, chemotherapy has proven to be highly successful in improving the lives of patients with cancer and in eradicating many forms of tumors (Palumbo et al. 2013). Despite the increased effectiveness and endurance of current therapies, multidrug resistance and the adverse effects of the long-term use of anticancer chemotherapy remain major challenges (Schirrmacher 2019; Hussain et al. 2019). Therefore, there is an urgent need for the development of effective anticancer drugs whose toxicity to normal tissues as well as acute and long-term side effects are minimized.

The chalcones scaffolds are flavonoid and isoflavonoid precursors, and they are ubiquitous in natural products such as citrus fruits, vegetables, and spices (Sahu et al. 2012; Zakaryan et al. 2017). Chalcones, both natural and synthetic analogs, have anticancer, anti-inflammatory, and antimutagenic activities. They have the potential to target molecules that are implicated in the beginning and progression of cancer (Jandial et al. 2014). Synthetic chalcone analogs display various biological activities influenced by the functional groups of the chalcone derivative. As previously mentioned, the methoxy alterations, depending on their position on the aryl rings (A and B), seem to affect the anticancer activity of chalcones. Chalcones are found in two isomers: trans (E) and cis (Z). The E isomer is the most stable, and thus the most prevalent, structure among the chalcones (Evranos Aksöz and Ertan 2011). Trimethoxy chalcone exerts anticancer activity in different human cancer cell lines, namely, ACHN, Panc 1, Calu 1, H460, and HCT116. Another study by Srinivasan et al. has reported that (E) trimethoxy phenyl suppresses NF-ΚB activation in A549 lung cancer cells (Srinivasan et al. 2009). Hence, methoxylated chalcone provides an attractive scaffold to study its anticancer effect. The quinazolinone chalcone derivative (QC) demonstrated antitumor activity both in vitro and in vivo. It stopped cancer cell lines including PC-3, Panc-1, Mia-Paca-2, A549, MCF-7, and HCT-116 from proliferating. QC caused apoptosis in HCT-116 cells, as shown by the production of apoptotic bodies, increased G0 cell fraction, loss of mitochondrial membrane potential (m), decrease of Bcl-2/BAX ratio, and the activation of caspase-9, caspase-3, and PARP-1 (poly-ADP Ribose polymerase) cleavage. Additionally, QC inhibited both Ehrlich ascites carcinoma (EAC) and Ehrlich solid carcinoma (ESC). QC was determined to be nontoxic since no animals died due to the effect of QC therapy (Wani et al. 2016). Lophirones B and C, dimeric chalcones extracted from the stem bark of Lophira alata, have anticancer, antimutagenic, and antioxidant properties. Particularly, Lophirone C has the best anticancer, antimutagenic, and antioxidant properties against EAC cells (Ajiboye et al. 2014).

ESC, an aggressive and fast-growing carcinoma, is one of the in vivo experimental models used to investigate prospective anticancer therapies. EAC first appeared spontaneously in a female mouse as breast adenocarcinoma. Ehrlich cancer cells can develop both ascites and solid forms, whether implanted intraperitoneally (IP) or subcutaneously (Vendramini-Costa et al. 2010). Several earlier research has employed Ehrlich solid carcinoma as a model for anti-cancer drugs (El-Shorbagy et al. 2019; Elbialy and Mohamed 2020; Monem et al. 2020; Sharawi 2020; Barhoi et al. 2021).

In our previous in vitro study, one of a series of novel synthesized chalcone derivatives, (Mohamed et al. 2018), was the chalcone derivative (*E*)-1-(8,9-dimethoxy-1-phenyl-1,5,6,10btetrahydro[1,2,4]triazolo[3,4*a*]isoquinolin3yl)3(3,4,5trimethoxyphenyl)prop-2-en-1-one (CHE).

CHE showed promising anticancer effects against cancer cell lines with different metastatic potentials, including MCF7, A549, HEPG2, and HCT116. Importantly, CHE showed no cytotoxic effects on normal melanocyte HFB4 cell lines. Gene expression analysis showed that CHE upregulated the BAX, p53, and caspase-3 genes and downregulated BCL2, MMP1, and CDK4. Also, flow cytometer analysis demonstrates that CHE induced cell growth arrest at the G1 phase, inhibiting cell cycle progression at the G1/S transition. However, because these promising anticancer effects of CHE were all in vitro, we designed this study to explore the in vivo anticancer activity of CHE. Using different doses of CHE on ESC-bearing mice, we explore the molecular mechanism(s) underlying the effects of CHE and compare them with those of the widely used chemotherapeutic doxorubicin (DOX).

## Materials and methods

### Chemicals and kits

Chemicals and kits employed in this study were as follows: DMSO (SERVA, Heidelberg, Germany, CAS:67–68-5), tween 80 (Rankem, Haryana, India, CAS:9005–65-6), doxorubicin (Sigma-Aldrich, Steinheim, Germany), Hank’s balanced salt solution (HBSS) (Biowest, Nuaillé, France, CAS: L0607-500), and RNA later (PUREGENE, Asia, PG-100153). GeneJET RNA Kit #k0731, RevertAid First Strand cDNA Kit #k1622, Maxima SYBR Green qPCR Master Mix (2X) #k1061, and GeneJET Genomic DNA Kit #k0721 were purchased from Thermo Scientific (Vilnius, Lithuania). Bio-diagnostic assay kit was from Bio diagnostic (Giza, Egypt), anti-mouse bax antibody (ABclonal, Woburn, UK, A12009), and anti-mouse ki67 antibody (Thermo Fisher Scientific, Cheshire, UK, #RB-9043-R7).

### CHE synthesis

**Fig. 1:**
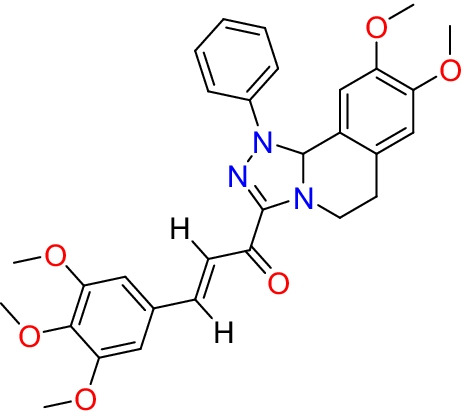
Molecular structure of chalcone derivative CHE, (E)-1-(8,9-dimethoxy-1-phenyl-1,5,6,10b-tetrahydro[1,2,4] triazolo[3,4-a]isoquinolin-3-yl)-3(3,4,5trimethoxyphenyl)prop-2-en-1-one

In our earlier research, we described the preparation of a chalcone derivative from 3-acetyl-8,9-dimethoxy-1-phenyl-1,5,6,10b-tetra-hydro[1,2,4] triazolo[3,4-a] isoquinoline (A), which in turn was obtained by reacting 3,4-dihydro-6,7-dimethoxyisoquinoline with nitrilimines (Elwan et al. 1996; Hassaneen et al. 2011). The chalcone derivative (*E*)-1-(8,9-dimethoxy-1-phenyl-1,5,6,10b-tetrahydro[1,2,4] triazolo[3,4-*a*]isoquinolin-3-yl)-3(3,4,5trimethoxyphenyl)prop-2-en-1-one was formed via Claisen–Schmidt condensation of compound A with an equimolar amount of substituted aldehydes using potassium hydroxide as a catalyst (Fig. 1).

### Animals

A total of 54 female BALB/c adult mice (6–8 weeks of age, 25 ± 3 g) were obtained from the animal facility of the National Cancer Institute at Cairo University (Giza, Egypt). Upon arrival, the mice were placed in plastic cages with sawdust bedding (five randomly selected mice per cage). Mice were given 1 week to acclimate in a normal laboratory environment (temperature in the 22–25 °C range, humidity, and a 12 h light/dark cycle) and had unrestricted access to a standard laboratory diet and water. All procedures used in the experiments were fully compliant with international standards for the care and management of laboratory animals. The experimental animal protocol (CU/I/F/54/19) has been approved by the Cairo University Institutional Animal Care and Use Committee (CU-IACUC).

### Determination of the maximum tolerated dose of CHE

Twenty-four healthy female mice, weighing 25 ± 3 g, were used to determine the maximum tolerated dose (MTD) and lethal dose (LD50) according to guideline no. 425 for the testing of chemicals of the Organization for Economic Co-operation and Development (OECD 2008). Four groups of six mice were housed and allowed to acclimatize for 7 days before the experimental procedure before they were injected with one dose of CHE IP. The body weight-adjusted doses were 2000 mg/kg (group 1), 1000 mg/kg (group 2), 550 mg/kg (group 3), and 450 mg/kg (group 4). Mice were monitored for mortality, weight loss, activity, urine, and changes in stool rates for 48 h. A dose–response curve was generated, plotting the % mortality versus dosage, and the correlation between the two was assessed via regression analysis.

### Induction of solid tumor in female mice and experimental design

EAC-bearing mice were sourced by the animal facility of the National Cancer Institute at Cairo University (Giza, Egypt). Mice were randomly divided into six groups of five mice. Each animal was intramuscularly implanted (in the thigh of the left hind limb) with a 200 µL tumor cell suspension in PBS containing approximately 2 × 10^6^ cells. After 8 days of inoculation, when the tumor was palpable, mice were treated IP with CHE twice per week for 2 weeks. CHE was dissolved in a mixture of 5% DMSO, 5% tween 80, and 90% H_2_O. DOX (4 mg/kg), used as a reference drug, was IP injected twice a week for 2 weeks (Quwaydir et al. 2019).

The groups were assigned as follows: group I, the negative control mice IP injected with a vehicle; group II, Ehrlich solid tumor-bearing mice subjected to IP injection of the vehicle; group III, Ehrlich solid tumor-bearing mice treated with 4 mg/kg DOX as a positive control group; group IV, Ehrlich solid tumor-bearing mice treated with 107 mg/kg CHE; group V, Ehrlich solid tumor-bearing mice treated with 214 mg/kg CHE; group VI, Ehrlich solid tumor-bearing mice treated with 321 mg/kg CHE. At the end of the experiment, we measured tumor weight (g) and relative tumor volume (RTV), defined by the formula RTV = Vf/Vi, where Vf denotes final tumor volume and Vi denotes initial tumor volume. We also measured tumor growth inhibition according to the formula TGI = 100 − (T/C × 100), where *T* and *C* represent the RTV of the treated and control groups, respectively.

### Sample collection

After 2 weeks of treatment, mice were euthanized by cervical dislocation under anesthesia. For histopathological examination, a part of tumor tissues, liver, and kidney was fixed in 10% neutral-buffered formalin. For molecular studies, another part of tumor tissues was preserved in RNAlater and stored at − 80 °C. The remaining tissues were snap-frozen in liquid nitrogen and preserved at − 80 °C.

### Histopathological examination

The formalin-fixed part of the tissue was dehydrated by passing it through an ascending series of ethyl alcohol. After that, the alcohol was removed from the tissue using xylene, and the tissue was embedded in paraffin. Serial tissue sections of 5 μm thickness were stained with hematoxylin and eosin and examined under the microscope by an expert pathologist in a blind protocol.

### Assessment of antioxidant capacity in tumor tissue

The total antioxidant capacity (TAC) of tumor tissue was determined using the Biodiagnostic test kit. Tumor tissue was homogenized in a cold potassium phosphate buffer (pH 7.4) composed of 5 mM potassium phosphate, 0.9% sodium chloride, and 0.1% glucose. The tissue lysate was centrifuged for 15 min at 4000 rpm, and the supernatant was mixed with the substrate (H_2_O_2_) and incubated at 37 °C for 10 min. Afterward, the chromogen and enzyme buffer were added, and the mixture was incubated at 37 °C for 5 min. The relative absorbance of samples thus prepared and blanks was measured at 505 nm against distilled H_2_O using a microplate reader (Infinite®200 PRO NanoQuant, Tecan; Männedorf, Zürich, Switzerland). Finally, TAC was calculated, in units of concentration, using the equation $\mathrm{TAC }\left[\mathrm{mM}\text{/L}\right] = \left({A}_{b}-{A}_{sa}\right)\times 3.33$, where $\mathrm{TAC}$ is the total antioxidant capacity and ${A}_{b} \mathrm{and }{A}_{sa}$ are the absorbances of the blank and the sample against distilled H_2_O, respectively.

### DNA fragmentation assay

Genomic DNA was extracted using the GeneJET Genomic DNA Purification Kit following the manufacturer’s instructions. The extracted DNA samples were pooled across animals within groups and electrophoresed on a 2% agarose gel using a reduced voltage to avoid overheating that would cause heat-induced confounds in DNA fragmentation. Finally, the fragmented DNA was captured and visualized using the BioSpectrum 815 Imaging System (UVP, CA, USA).

### Comet assay (single-cell gel electrophoresis)

We used an alkaline comet assay to evaluate and detect alkali labile sites and DNA double- and single-strand breaks, as previously described (Tice et al. 2000; Dhawan et al. 2003). Briefly, tumor tissue (approximately 50 mg) was minced in Hank’s Balanced Salt Solution supplemented with 20 mM EDTA and 10% DMSO. Then, 10 µL of the clear layer was mixed gently by pipetting with 75 µL of 1% low melting point agarose and incubated at 37.5 °C for 5 min. The mixture was gently distributed on slides precoated with 1% agarose and incubated in freshly prepared lysing solution (2.5 M NaCl, 100 mM EDTA, and 10 mM Trizma base [pH 10], 1% Triton X-100, and 10% DMSO) for 24 h. Subsequently, the slides were incubated in electrophoresis buffer (pH > 13) for 30 min before the samples were electrophoresed by applying 0.74 V/cm and 300 mA for 30 min. Slides were removed from the buffer gently and drained on a tray and then neutralized using neutralization buffer (pH = 7.5) twice for 5 min each. Slides were dehydrated in absolute ethanol, stained with ethidium bromide, and visualized using a fluorescent microscope (Leica City, Germany). Microscopic images were analyzed using commercial software (CometScore V2.0). Tail length, tail DNA %, and tail olive moment scores were used to quantify DNA damage.

### Gene expression using reverse transcription-quantitative real-time PCR (RT-qPCR)

Total RNA was isolated using the GeneJET RNA Purification Kit following the manufacturer’s instructions. RNA concentration and purity were assessed by absorbances measured at 260 and 280 nm using the Infinite®200 PRO NanoQuant (Tecan). cDNA was synthesized using the RevertAid First Strand cDNA Synthesis Kit in the Veriti™ 96-Well Thermal Cycler following the manufacturer’s instructions. To quantify gene expression levels, SYBR Green qPCR maxima were used to amplify sequences specific to the gene of interest using the StepOnePlus™ Real-Time PCR System (Applied Biosystem). The primer sequences used in this study were designed using Primer3 software and synthesized by Vivantis Technologies (Selangor, Malaysia). They are depicted in Table 1. Relative gene expression was calculated using the fold change formula 2^−ΔΔCT^. Two housekeeping genes, *B2m* and *β-actin*, were evaluated for their stability using a web-based tool, and the most stable internal control was chosen according to the best keeper ranking as previously described (Pfaffl et al. 2004).

**Table 1:** The primer sequences used in quantitative real-time PCR

Gene name	Sequences		Product size (bp)
*B2m*	F	ACAGTTCCACCCGCCTCACATT	105
	R	TAGAAAGACCAGTCCTTGCTGAAG	
*β-actin*	F	GCAGGAGTACGATGAGTCCG	74
	R	ACGCAGCTCAGTAACAGTCC	
*Trp53*	F	CCCCTGTCATCTTTTGTCCCT	137
	R	AGCTGGCAGAATAGCTTATTGAG	
*Bax*	F	GTCTCCGGCGAATTGGAGAT	100
	R	ACCCGGAAGAAGACCTCTCG	
*Bcl2*	F	CATCGCCCTGTGGATGACTG	95
	R	GGCCATATAGTTCCACAAAGGC	
*Casp3*	F	GGAGTCTGACTGGAAAGCCGAA	113
	R	CTTCTGGCAAGCCATCTCCTCA	

### Immunohistochemical (IHC) staining for Ki67 and Bax

Formalin-fixed paraffin-embedded tissue sections were deparaffinized in xylene two times, 5 min each, followed by hydration by passage through a descending series of alcohol for 5 min each step. Heat-induced antigen retrieval was performed in a steamer chamber using citrate buffer (pH = 6.1). Slides were then allowed to cool and washed with distilled H_2_O three times for 5 min each, before being immersed in a hydrogen peroxide bath for 10 min to block the endogenous peroxidase activity. Then, the slides were rinsed off twice with distilled H_2_O and once with tris-buffered saline containing 0.1% tween (TBST, pH = 7.6) for 5 min. To inhibit nonspecific binding sites, the slides were treated in 1% bovine serum albumin (BSA)/TBST at room temperature for 1 h. Subsequently, slides were incubated with the primary antibodies against BAX diluted at 1:100 and ki67 diluted at 1:300 in 1% BSA/TBST at 4 °C overnight. Slides were washed three times in TBST, for 5 min each. Slides were washed with a chromogen solution, prepared by adding one drop of 3,3′ diaminobenzidine (DAB +) and substrate buffer, for 3 min, and then immersed in distilled water to stop the color reaction, followed by counterstaining with hematoxylin for 1 min. Finally, slides were cleared in xylene, dehydrated by passing through an ascending series of alcohol, and mounted with DPX. Slides were photographed using a light microscope (Olympus, Tokyo, Japan), and the fractional (%) stained area was calculated using Fiji ImageJ software version 1.53 h.

### Statistical analysis

Data analyses were conducted using the Statistical Package for the Social Sciences (version 25). The sample size required for statistical power was calculated using Gpower 3.1 software. Tests for outlier values were conducted using Minitab (version 17). A normality test was conducted using skewness and kurtosis, and the normally distributed data were analyzed through parametric tests. The statistical significance levels for the difference between the two groups were determined using Student’s *t*-test. To determine the significance of the difference between two groups where there were multiple comparisons, we used the one-way analysis of variance (ANOVA) followed by the Tukey post hoc test. The correlation of the two variables was evaluated with Pearson’s correlation. Data were expressed as mean ± SEM; a *p*-value of ≤0.05 was considered significant for all tests. Graphs were generated using GraphPad Prism 8.

## Results

### MTD and LD50 determination

**Fig. 2:**
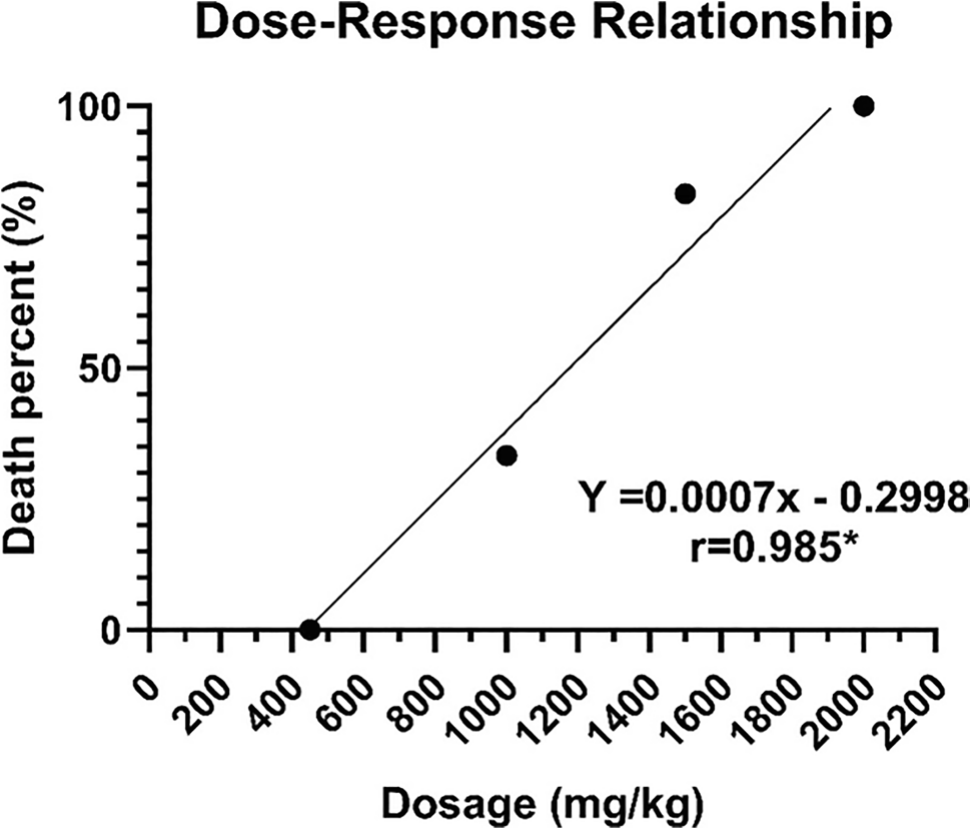
Dose–response curve of CHE measured 48 h after intraperitoneal injection in mice with Ehrlich tumor

First, we determined MTD and LD50 of CHE, by monitoring the animals for 48 h after a single IP injection of CHE. The dose–response relationship curve (Fig. 2) shows a linear dependence of % lethality on the dose administered, with a significant correlation coefficient (*r* = 0.9705, *P* < 0.05). MTD = 428 mg/kg and LD50 = 1142 mg/kg were determined by extrapolating the regression to 0 and 50% lethality, respectively. Based on this analysis, we determined the doses of 107, 214, and 321 mg/kg (at 25%, 50%, and 75% of MTD, respectively) to be administered in further experiments.

### CHE treatment retards ESC growth and reduces RTV

**Fig. 3:**
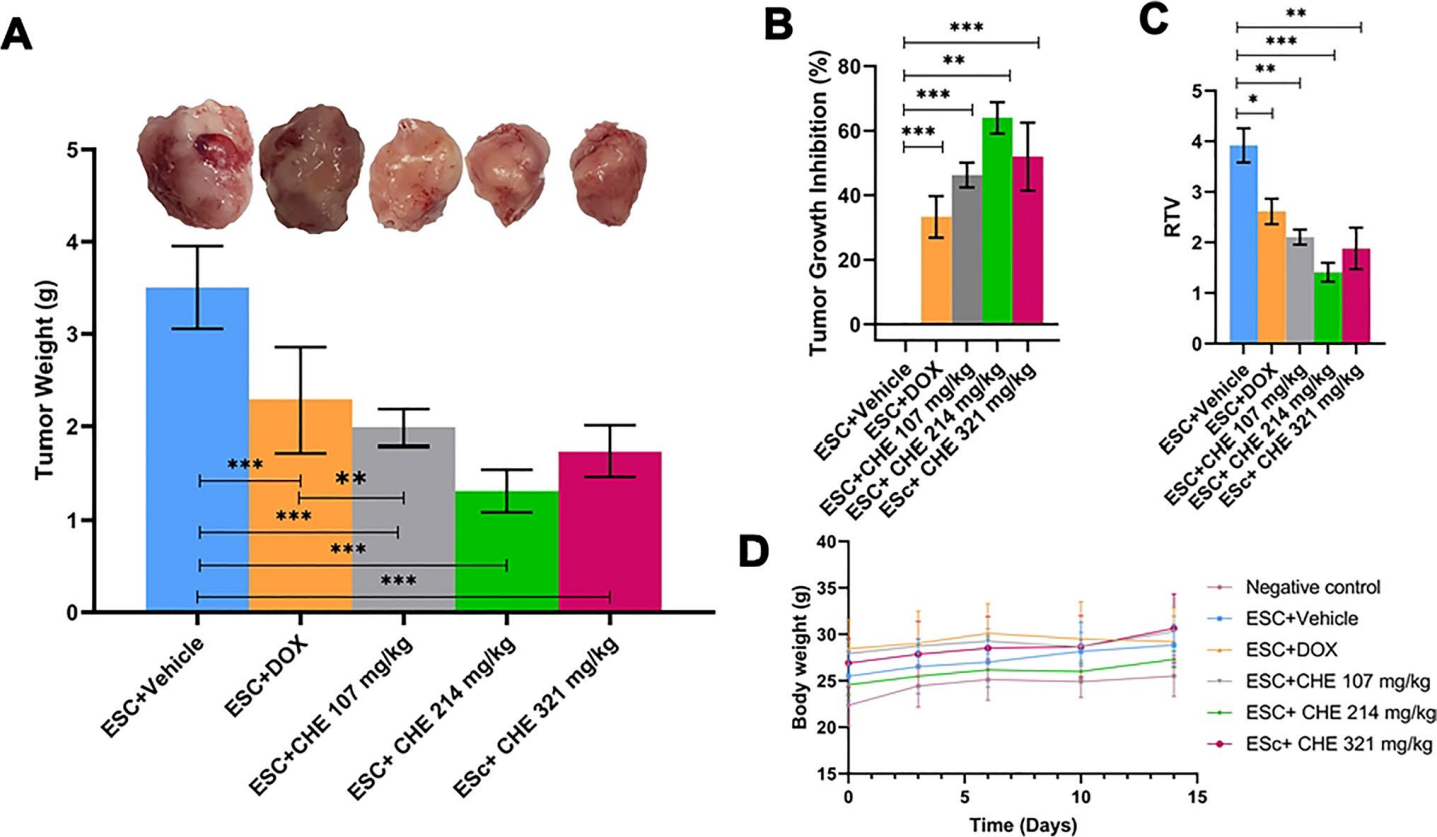
CHE treatment attenuates ESC growth in mice. ESC-tumor-bearing mice were treated with doses of CHE at 107, 214, and 321 mg/kg twice a week for 2 weeks before tumor weight was assessed. Mice treated with the vehicle are the negative control, and those treated with doxorubicin (DOX) are a positive control. **A** ESC tumors weigh in each treatment group on day 14, with representative images of ESC tumors excised. **B** The fractional (%) inhibition of tumor growth. **C** The relative tumor volume (RTV). **D** The changes in body weight during the experimental period. Bars represent means ± SEM, *n* = 5. *P < 0.05, **P < 0.01, and ***P < 0.001, as determined via one-way ANOVA followed by Tukey’s multiple comparison test

At the end of the experiment, CHE and/or DOX treatment resulted in a significant reduction in tumor weight in all treatment regimens (Fig. 3A). Particularly, relative to tumors in the negative control group, treatment by ESC + DOX reduced tumor growth by 33.2% (*P* < 0.001), ESC + CHE 107 mg/kg by 46.2% (*P* < 0.001), ESC + CHE 214 mg/kg by 63.9% (*P* < 0.01), and ESC + CHE 321 mg/kg by 51.9% (*P* < 0.001) (Fig. 3B). RTV was also significantly decreased in all treatment groups (*P* < 0.05 for ESC + DOX, *P* < 0.01 for ESC + CHE 107 mg/kg and ESC + CHE 321 mg/kg, and *P* < 0.001 for ESC + CHE 214 mg/kg), in comparison with ESC + vehicle group (Fig. 3C). Notably, the body weight was increased normally in each group except for the DOX-treated group, which showed a decreased total body weight by the end of the 2-week treatment (Fig. 3D**)**.

### Histopathology

**Fig. 4:**
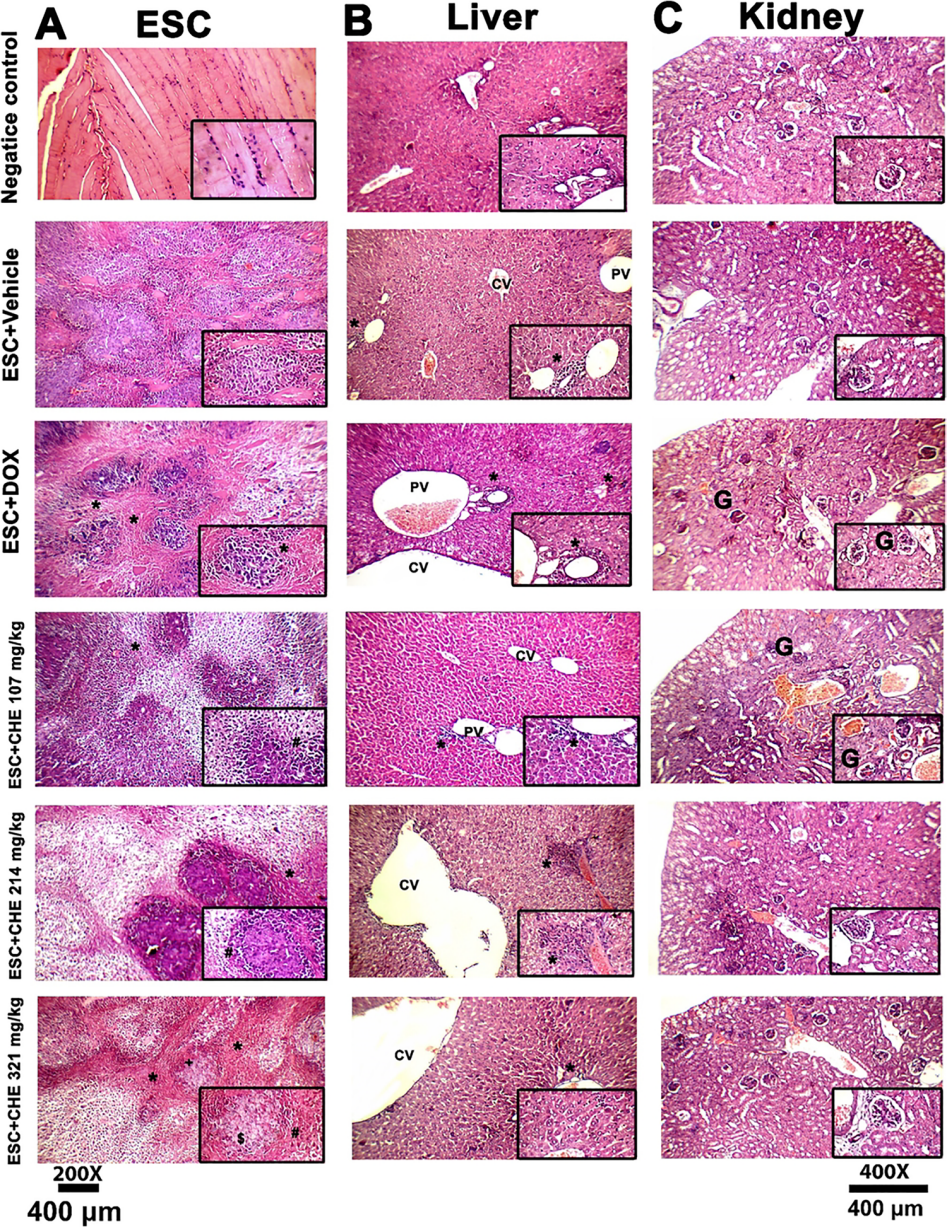
Histopathological sections of tumor, liver, and kidney tissues from either negative control or ESC-bearing mice treated with DOX and different doses of CHE (107, 214, and 321 mg/kg). **A** Photomicrographs of tumor tissue showing ( +) necrotic nodules of tumor cells, ($) nodules composed mainly of ghost cells, (#) marked apoptosis, and (*) large areas of perinodular necrosis with few karyorrhectic fragments in the treated groups. **B** Photomicrographs of liver tissue with (*) mild inflammatory infiltrate, (P) mild dilation in the portal, and (CV) central vein. **C** Photomicrographs of kidney tissue showing (#) mildly congested glomeruli in the ESC + DOX and ESC + CHE 107 mg/kg groups with normal renal capsule, normal glomeruli, normal Bowman’s spaces, normal proximal tubules with preserved brush border, and normal collecting tubules in the treated groups. H&E 200 × , the inset (black rectangle) image is at × 400 magnification

Evaluation of tumor tissues in the ESC + vehicle group revealed several pleomorphic cells with hyperchromatic nuclei penetrating between muscles and fat, a few scattered apoptotic cells, distributed mitotic figures, scattered giant cells, and small regions of perinodular necrosis. Relative to the ESC + DOX group, treatment with CHE led to a high regression of tumor growth associated with small, partially necrotic nodules of tumor cells composed mainly of ghost cells, marking apoptosis, and large areas of perinodular and intranodular necrosis with few karyorrhectic fragments (Fig. 4A).

The liver of the negative control group was found to have a normal structure, with normal portal veins, normal bile ducts, normal hepatocytes in the periportal region, and normal central veins surrounded by hepatocytes. The effect of CHE on the liver showed a dilation in the central vein with moderately dilated congested portal veins and normal hepatocytes and mild intralobular inflammatory infiltrate with few scattered apoptoses in the high CHE dose (ESC + CHE 321 mg/kg) group. In the ESC + DOX group, mild portal inflammatory infiltrates, moderately dilated congested portal veins, dilated bile ducts, markedly dilated central veins, mild intralobular inflammatory infiltrates, scattered apoptosis in the perivenular area, and microvesicular steatosis of hepatocytes more marked in the perivenular area were observed. Interestingly, the liver tissues from the ESC + CHE 107 mg/kg animals showed a minimal effect of CHE (Fig. 4B).

In the treated groups, histopathological examination revealed normal kidney structure with normal renal capsule, normal glomeruli, normal Bowman’s capsules, normal proximal tubules with preserved brush border, and normal collecting tubules, except for the ESC + DOX and ESC + CHE 107 mg/kg groups, in which glomeruli were mildly congested. This observation reflects the minimal harmful effect of CHE on kidney tissues (Fig. 4C).

### CHE induces oxidative stress and DNA damage in tumor tissues

**Fig. 5:**
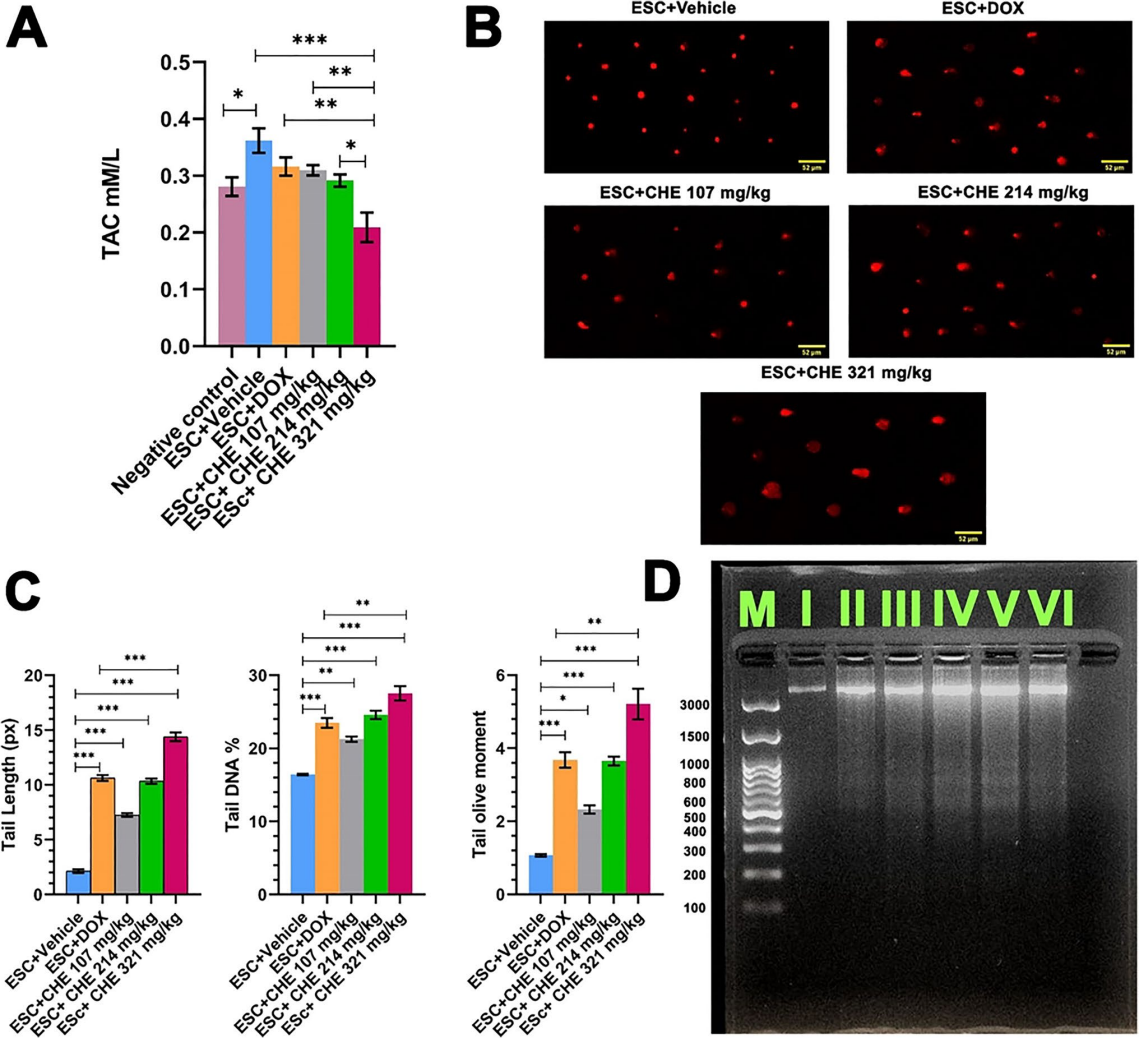
The effect of chalcone derivative on total antioxidant capacity (TAC) and DNA damage in ESC tumor tissues. **A** CHE treatment reduces TAC in tumor tissues. **B** Representative images of damaged DNA induced by CHE or DOX treatments of ESC tumor tissue compared with intact DNA of the negative control group. **C** Quantification of DNA damage parameters (tail length, % tail DNA, and tail moment) in the treatment groups. In each sample, 50 or more cells were analyzed. CometScore software (V2.0) was used to assess DNA damage parameters. Bars represent means ± SEM, *n* = 3. *P < 0.05, **P < 0.01, and ***P < 0.001, as determined via one-way ANOVA followed by Tukey’s multiple comparison test. **D** Representative photograph showing DNA fragmentation of DNA extracted from the tumor or healthy tissues of the different experimental treatment groups. Lanes M: DNA ladder, I: negative control group, II: ESC + vehicle group, III: ESC + DOX group, IV: ESC + CHE 107 mg/kg group, V: ESC + CHE 214 mg/kg group, VI: ESC + CHE 321 mg/kg group

Since multiple chalcone derivatives have an influence on the oxidative status in different experimental models of cancer (Wang et al. 2020; Huang et al. 2020; Guan et al. 2021; Khusnutdinova et al. 2021), we assessed the oxidative stress level in tumor tissue by measuring TAC upon CHE treatment. TAC in the ESC + Vehicle group was significantly increased (*P* < 0.05) in comparison with the negative control group. Furthermore, ESC + CHE 321 mg/kg group showed a significant depletion in TAC compared with ESC + vehicle group (*P* < 0.001), ESC + DOX group (*P* < 0.01), ESC + CHE 107 mg/kg group (*P* < 0.01), and ESC + CHE 214 mg/kg group (*P* < 0.05) (Fig. 5A).

We next examined whether CHE treatment had the potential to induce DNA damage. For this purpose, an alkaline comet assay was conducted. DNA damage was assessed based on three parameters, namely, tail length, tail DNA %, and tail olive moment. Comet assay results revealed that CHE treatment significantly increased DNA damage in tumor tissue in a dose-dependent manner relative to the ESC + vehicle group (Fig. 5B and C). Tail length was significantly increased (*P* < 0.001) in all treated groups compared with the ESC + Vehicle group. Notably, ESC + CHE 321 mg/kg group exhibited a significant increase (*P* < 0.001) in tail length compared with the ESC + DOX group. Tail DNA % was significantly increased over the levels in the negative control in the ESC + DOX (*P* < 0.001), ESC + CHE 107 mg/kg (*P* < 0.01), ESC + CHE 214 mg/kg (*P* < 0.001), and ESC + CHE 321 mg/kg (*P* < 0.001) groups. Interestingly, ESC + CHE 321 mg/kg group showed a significant increase (*P* < 0.01) in tail DNA % compared with the ESC + DOX group. Similarly, the tail olive moment was significantly increased in each treatment group relative to the negative control (ESC + DOX, *P* < 0.001; ESC + CHE 107 mg/kg, *P* < 0.05; ESC + CHE 214 mg/kg, *P* < 0.001; ESC + CHE 321 mg/kg, *P* < 0.001). Moreover, the tail olive moment was significantly increased in the ESC + CHE 321 mg/kg group compared with the ESC + DOX group (*P* < 0.01).

For further confirmation of the DNA damage-inducing activity of CHE, DNA was extracted from tumor tissues from all groups and subjected to 2% agarose gel electrophoresis. Our results showed remarkable DNA fragmentation in all treated groups compared with the untreated control group (Fig. 5D).

### CHE affects the expression of apoptosis-related genes in tumor tissues

**Fig. 6:**
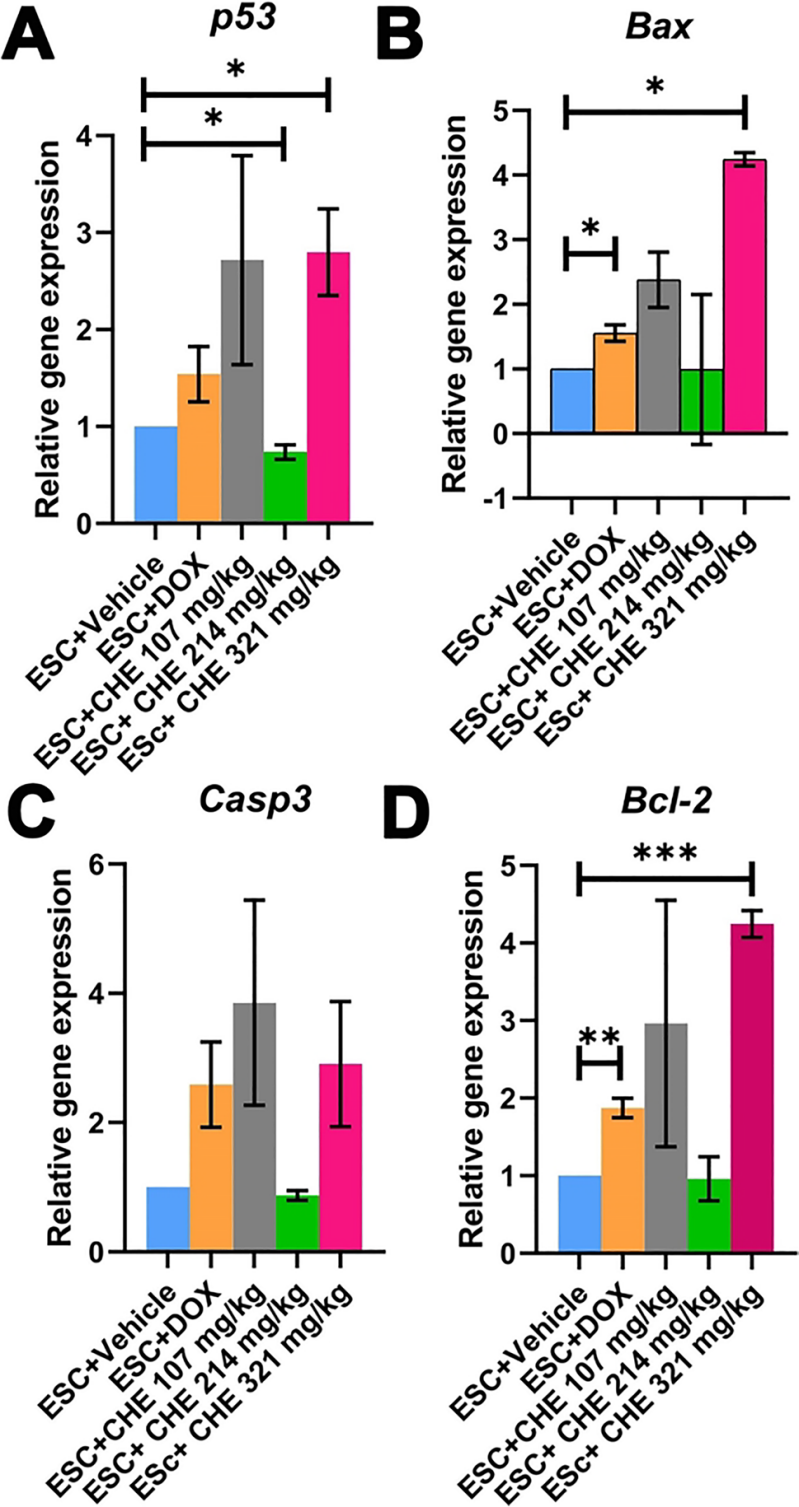
Effect of CHE treatment on mRNA expression levels of the apoptosis-related genes *p53*, *Bax*, *Casp3*, and *Bcl2* assessed by *qPCR* in tumor tissues of ESC-bearing mice. Bars represent means ± SEM, *n* = 3. *P < 0.05, **P < 0.01, and ***P < 0.001, as determined via Student’s *t*-test

Since CHE induced DNA damage, which in turn may affect apoptosis, qPCR was performed to quantify mRNA expression levels for four apoptosis-related genes (*p53*, *Bax*, *Casp3*, and *Bcl-2*). *B2m* gene was chosen as the internal housekeeping gene according to best keeper analysis compared with *β-actin*, as shown in **(**Fig. A.1**)**. The results of qPCR revealed that *p53* mRNA expression levels were significantly (by 2.8-fold) upregulated in tumor tissues of the highest dose CHE treatment (ESC + CHE 321 mg/kg) group relative to the vehicle-treated group (*P* < 0.05) (Fig. 6A). *Bax* mRNA expression levels were significantly upregulated in the ESC + DOX group (by 1.5-fold; *P* < 0.05) and ESC + CHE 107 mg/kg group (by 4.2-fold; *P* < 0.05) relative to the vehicle-treated group (Fig. 6B). *Casp3* mRNA expression levels were upregulated in ESC + DOX, ESC + CHE 107 mg/kg, and ESC + CHE 321 mg/kg groups relative to the vehicle-treated group but did not reach significance (Fig. 6C). *Bcl-2* mRNA expression levels were upregulated by 1.8-fold (*P* < 0.01) in the ESC + DOX group and 4.2-fold (*P* < 0.001) in the ESC + CHE 321 mg/kg group relative to the vehicle-treated group (Fig. 6D).

### CHE reduces ki67 and increases BAX protein expression levels

**Fig. 7:**
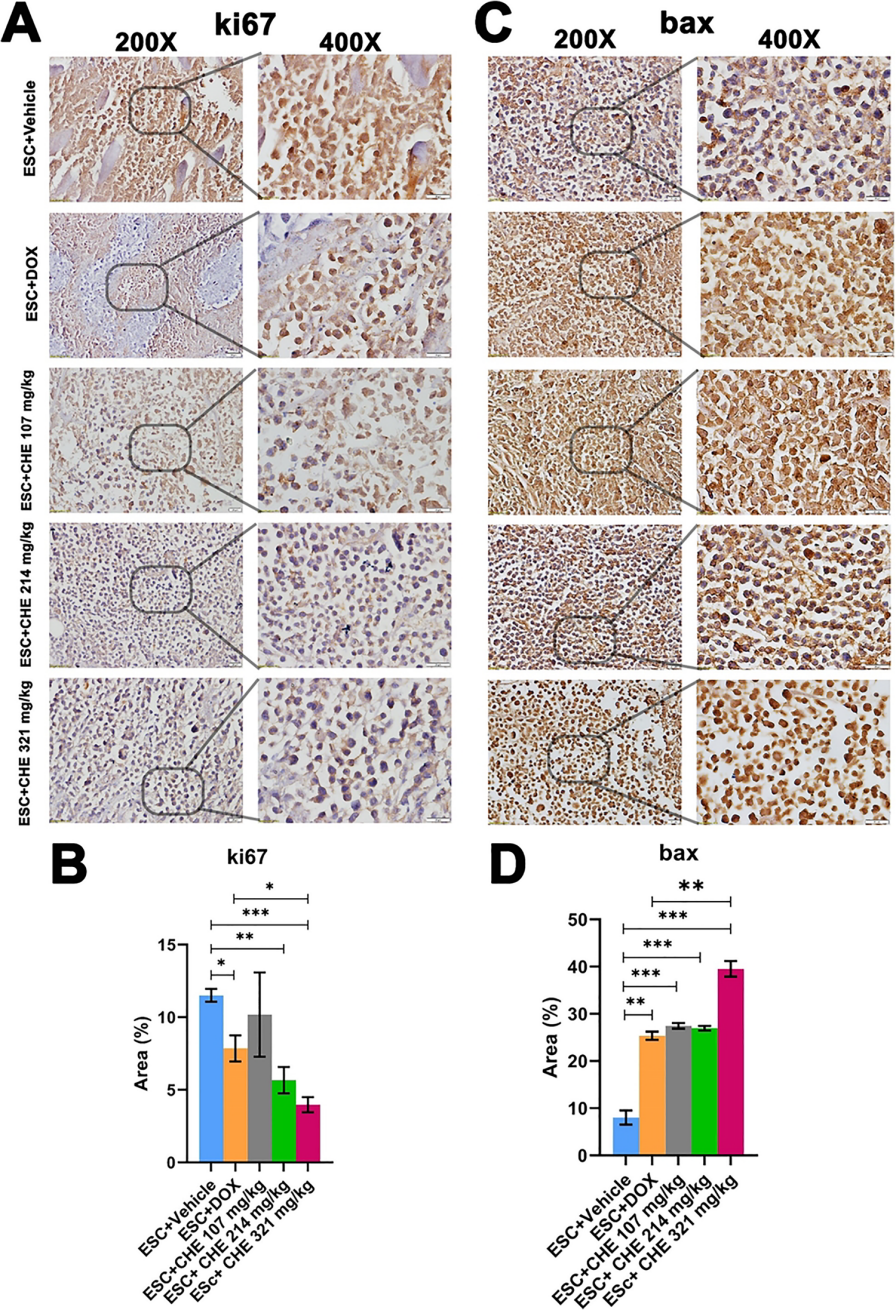
Immunohistochemical evaluation of the proliferation marker ki67 and the pro-apoptotic marker BAX in tumor tissues obtained from animals in each treatment group. **A** Representative microscopic images (× 200 on the left and 400 × blow-up of marked areas on the right) of immunostaining (brown color) of ki67 in tumor tissues. **B** Quantitative analysis of the fraction of area containing positive ki67 immunostaining. **C** Same as A but immunostaining for BAX. **D** Quantitative analysis of the fraction of area containing positive BAX immunostaining. Area fraction was calculated using ImageJ software (Fiji). Bars represent means ± SEM, *n* = 3. *P < 0.05, **P < 0.01, and ***P < 0.001, as determined via Student’s *t*-test

The expression pattern of the proliferative index marker ki67 and the apoptosis-related BAX was examined via immunohistochemistry. Our data analysis revealed that tumor tissues of the ESC + vehicle group displayed high positive staining for nuclear ki67 as measured by the fractional stained area (11.5 ± 0.44%) (Fig. 7A and B). By contrast, ki67 expression was significantly decreased in groups ESC + DOX (7.85 ± 0.68%, *P* < 0.05), ESC + CHE 214 mg/kg (5.66 ± 0.49%, *P* < 0.01), and ESC + CHE 321 mg/kg (3.97 ± 0.34%, *P* < 0.001). Of note, tumor tissues of the highest CHE dosage group (ESC + CHE 321 mg/kg group) had a significantly lower ki67 immunostaining (*P* < 0.05) than those of the ESC + DOX group (Fig. 7 A and B). BAX expression resulted in a week of staining in the negative control (8.02 ± 1.48%; ESC + vehicle). By contrast, BAX expression was significantly stronger in tumor tissues of all treatment groups (ESC + DOX, 25.36 ± 0.87%, *P* < 0.01; ESC + CHE 107 mg/kg, 27.45 ± 0.59%, *P* < 0.001; ESC + CHE 214 mg/kg, 26.92 ± 0.50%, *P* < 0.001; and ESC + CHE 321 mg/kg, 39.53 ± 1.63%, *P* < 0.001) than in the negative control. Intriguingly, BAX expression in tumor tissues of the highest CHE dosage group (ESC + CHE 321 mg/kg) was significantly increased (*P* < 0.01) compared with the DOX treatment (ESC + DOX) group (Fig. 7 C and D).

## Discussion

Our study was conducted to investigate the anticancer properties of CHE, a newly synthesized chalcone derivative, which has a documented potent anticancer effect in vitro against different cancer cell lines MCF7, A549, HCT116, and HepG2 (Mohamed et al. 2018). This study was designed to further evaluate the anticancer activity of CHE in vivo using Ehrlich solid tumor. Our data demonstrate that CHE regressed tumor weight and induced oxidative stress and DNA damage, which may ultimately result in apoptosis.

One of the features of cancer cells is increased aerobic glycolysis, which is combined with high levels of oxidative stress (Cairns et al. 2011) caused by reactive oxygen species (ROS) that build up because of an imbalance between ROS production and removal. Changes in various signaling pathways that impact cellular metabolism lead to elevated ROS levels in cancer cells (Diehn et al. 2009; Sznarkowska et al. 2017). Cancer cells are aided in the reduction of ROS levels by enhanced antioxidant defense mechanisms that help them acclimatize to the redox imbalances caused by fast growth (Jones and Thompson 2009). Thus, oxidative stress, through the increased amounts of ROS that is the driver of cell damage, may inhibit tumor growth. The increased ROS causes cancer cells to activate their robust antioxidant systems to overcome such stress. TAC is a parameter to assess the ability of the cancer cells to counteract oxidative stress (Trachootham et al. 2009). This feature provides an intriguing window of opportunity for therapeutic intervention since cancer cells may be more susceptible to drugs that induce increased ROS generation than normal cells (Gorrini et al. 2013). Indeed, CHE treatment caused a significant decrease in TAC in cancer cells, indicating an elevated ROS level associated with the induction of oxidative stress. This finding is in line with other reports that chalcone induced oxidative stress in chronic myelogenous leukemia (k562) cancer cells (Li et al. 2019), human glioma cell line U87-MG, and in a xenograft model in vivo (Loch-Neckel et al. 2015), and human colorectal HCT116 cells led to DNA damage and apoptosis (Takac et al. 2020).

Additionally, we proved the potency of CHE as an inducer of DNA damage. CHE treatment showed a remarkably significant increase in all DNA damage parameters and DNA fragmentation patterns, as evidenced via DNA fragmentation and comet assays. Other studies using different chalcones compounds unveiled their DNA damaging potency in vitro and in vivo. As reported previously, chalcone derivatives induced apoptosis and DNA damage by raising ROS levels in melanoma cells (Li et al. 2020). Similar results were observed for trimethoxy chalcone in A549 human lung cancer cells (Gil et al. 2019). In addition to their effect on oxidative stress-induced DNA damage, chalcones can cause DNA damage by binding DNA strands through van der Waal forces and aromatic ring stacking interactions. The unsaturated carbonyl system in chalcone compounds supports stronger electrostatic interactions between the hydrogen and DNA bases, as evidenced via molecular docking experiments showing that chalcones are bound to a DNA dodecamer with many hydrogen bonds (El-Wakil et al. 2020). This may further explain DNA damage induction by chalcone derivatives.

It is known that p53 becomes active in response to DNA damage, with its capacity to bind DNA and induce transcriptional activation increasing as its expression levels are rising quickly (Lakin and Jackson 1999). p53 stimulates target genes, resulting in DNA damage repair, cell growth inhibition, and apoptosis. Particularly, when DNA damage is severe, p53 triggers the activation of pro-apoptotic genes such as *Bax*, resulting in programmed cell death (Crowe and Sinha 2006). Consistent with those findings, CHE in our study significantly upregulated the expression of *p53* and *Bax* mRNA levels and BAX protein levels in response to DNA damage, eventually leading to apoptosis that we observed via histopathological examination in tumor tissue. Apoptosis induction by chalcones via upregulating *p53* and *Bax* expressions was reported in previous studies using different experimental models (Hsu et al. 2005, 2006; Singh et al. 2014; Loch-Neckel et al. 2015; Bagul et al. 2017; Cabral et al. 2017; Fong et al. 2017; Kim et al. 2017). Surprisingly, both DOX and CHE increased *Bcl-2* mRNA expression in tumor tissues of treated mice groups. This may be attributed to ROS via promoting phosphorylation and ubiquitination of proteins in the *Bcl-2* family, resulting in elevated pro-apoptotic protein levels and reduced antiapoptotic protein levels (Li et al. 2004). Also, docking studies revealed that chalcone compounds could inhibit the BH3 domain in BCL2 protein, thus inhibiting the antiapoptotic activity of BCL2 (Dey et al. 2020). Together, these findings suggest that CHE may inhibit *Bcl-2* activity, although its mRNA expression levels were increased.

Interestingly, CHE treatment significantly decreased the proliferative marker Ki67. The Ki67 protein has been extensively studied at the molecular level, and it has long been used as a prognostic and predictive marker in cancer diagnosis and treatment. (Li et al. 2015). Decreased levels of Ki67 upon treatment with chalcones are reported in several studies. For example, Maioral M et al. reported a chalcone-induced decrease in Ki67 in K562 and Jurkat cells. Another investigation using human non-small-cell lung cancer found that cardamonin treatment resulted in a reduction in Ki67 expression, and another study found that Ki67 was decreased in murine B16 melanoma cells in C57/BL6 mice upon treatment by xanthohumol chalcone. Chalcone also decreased the Ki67 in an in vivo model of triple-negative breast cancer cells (Luo et al. 2021).

Likely acting via some or all of the above molecular action pathways, CHE eventually caused a significant decrease in tumor weight, relative tumor size, and a significant tumor growth inhibition in CHE-treated mice compared with mice in the vehicle-treated group and was more effective than the reference drug DOX. One limitation that could be associated with this study is that CHE induces DNA damage, which may have an influence on normal organs. However, our previous results showed that CHE had no cytotoxic effect on the normal melanocytes (HFB4) cells. In addition, using docking simulation studies, CHE has a high binding affinity for EGFR and DHFR, which are overexpressed in cancer cells. Consistently, our histopathological examination revealed a minimal cytotoxic effect of CHE on liver and kidney tissues. This minimal effect may be potentially fully eliminated in future studies using synergistic treatment with strong antioxidant compounds to reduce the elevated ROS levels in the liver and kidney. One example is N-acetyl cysteine, which was shown to reduce the ROS effect of Zn oxide nanoparticles in the liver and kidney tissues without affecting its antitumor effect (El-Shorbagy et al. 2019).

## Conclusion

Based on the results of our study, we can infer that CHE treatment is effective against ESC in mice. CHE exerts a promising anticancer activity against ESC via the depletion of TAC with subsequent DNA damage, triggering the upregulation of the pro-apoptotic genes such as *p53* and *Bax*. Moreover, CHE decreased the proliferative marker Ki67 and increased BAX protein in tumor tissues. Overall, CHE may emerge as a potential therapy for solid tumors with minimal toxicity to vital organs. Further studies are needed to improve the selective delivery of CHE to cancer cells using nanoparticle-based delivery systems that would enable lowering the dose and increasing the therapeutic potency of CHE.

## Supplementary Information

Below is the link to the electronic supplementary material.

Supplementary file1 (DOCX 27 KB)

Supplementary file2 (PZFX 8 KB)

Supplementary file3 (PZFX 9 KB)

Supplementary file4 (PZFX 9 KB)

Supplementary file5 (PZFX 10 KB)

Supplementary file6 (PZFX 9 KB)

Supplementary file7 (PZFX 11 KB)

Supplementary file8 (PZFX 17 KB)

Supplementary file9 (PZFX 21 KB)

Supplementary file10 (PZFX 14 KB)

Supplementary file11 (PDF 151 KB)

Supplementary file12 (PDF 843 KB)

Supplementary file13 (PDF 719 KB)

## Data Availability

Data available on request.
