# Supplementary figures and images for: [1,2,4] Triazolo [3,4-a]isoquinoline chalcone derivative exhibits anticancer activity via induction of oxidative stress, DNA damage, and apoptosis in Ehrlich solid carcinoma-bearing mice
Source: Naunyn Schmiedebergs Arch Pharmacol. 2022 Jul 26;395(10):1225–38. doi: 10.1007/s00210-022-02269-5 (PMC9467967; doi:10.1007/s00210-022-02269-5)

# Supplementary figures


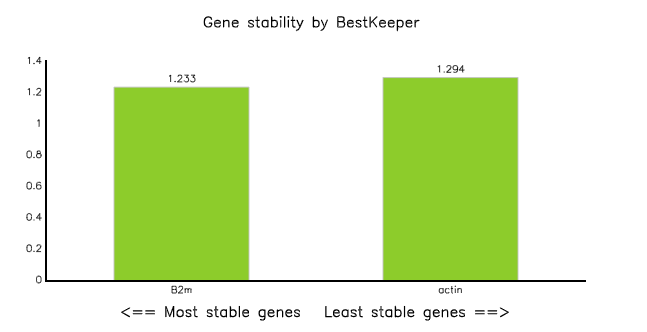


**Fig. A.1.** Gene stability of internal reference control by best keeper.

Supplement: Supplementary file 1 — Supplementary file1 (DOCX 27 KB) [file 210_2022_2269_MOESM1_ESM.docx]
