# Supplementary material for: [1,2,4] Triazolo [3,4-a]isoquinoline chalcone derivative exhibits anticancer activity via induction of oxidative stress, DNA damage, and apoptosis in Ehrlich solid carcinoma-bearing mice
Source: Naunyn Schmiedebergs Arch Pharmacol. 2022 Jul 26;395(10):1225–38. doi: 10.1007/s00210-022-02269-5 (PMC9467967; doi:10.1007/s00210-022-02269-5)
Supplement: Supplementary file 11 — Supplementary file11 (PDF 151 KB) [file 210_2022_2269_MOESM11_ESM.pdf]

enago
Author First, Quality First
www.enago.com

ulatus
localize to globalize
www.ulatus.com

voxtab
we are all ears
www.voxtab.com

CERTIFICATE OF EDITING

This is to certify that the paper titled [1,2,4]Triazolo[3,4-a]isoquinoline Chalcone Derivative Exhibits Anticancer Activity via Induction of Oxidative Stress, DNA Damage, and Apoptosis in Mice with Ehrlich Solid Carcinoma commissioned to us by Amr Ahmed Abdelfattah Ahmed WalyEldeen has been edited for English language, grammar, punctuation, and spelling by Enago, the editing brand of Crimson Interactive Inc. under Copyediting/Language editing.

- ISO 17100:2015
  Translation Service Providers
- ISO 27001:2013
  Information Security Management System
- ISO 9001:2015
  Quality Management System

Issued by:

Enago, Crimson Interactive Inc.
160, Greentree Dr, Ste 101 street,
Dover City, Kent, Delaware, 19904
Phone: +1-302-498-8358

Disclaimer : The intent of the author's message has been preserved during the editing process. The author is free to accept or reject our changes in the document after reviewing our edits. This certificate has been awarded at the time of sharing the final edited version (full file or sections of the file) with the author. Enago does not bear any responsibility for any alterations done by the author to the edited document post .

**Japan**	www.enago.jp, www.ulatus.jp, www.voxtab.jp
**Taiwan**	www.enago.tw, www.ulatus.tw
**China**	www.enago.cn, www.ulatus.cn
**Brazil**	www.enago.com.br, www.ulatus.com.br
**Germany**	www.enago.de
**Russia**	www.enago.ru
**Arabic**	www.enago.ae
**Turkey**	www.enago.com.tr
**S. Korea**	www.enago.co.kr
**Global**	www.enago.com, www.ulatus.com, www.voxtab.com

About Crimson:
Crimson Interactive INC is one of the world's leading academic research support services. Since 2005, we've supported over 2 million researchers in 125 countries with their publication goals.
