# Supplementary material for: [1,2,4] Triazolo [3,4-a]isoquinoline chalcone derivative exhibits anticancer activity via induction of oxidative stress, DNA damage, and apoptosis in Ehrlich solid carcinoma-bearing mice
Source: Naunyn Schmiedebergs Arch Pharmacol. 2022 Jul 26;395(10):1225–38. doi: 10.1007/s00210-022-02269-5 (PMC9467967; doi:10.1007/s00210-022-02269-5)
Supplement: Supplementary file 12 — Supplementary file12 (PDF 843 KB) [file 210_2022_2269_MOESM12_ESM.pdf]

# Institutional Animal Care and Use Committee (CU-IACUC)
# Cairo University

Cairo University

## *Approval to use animals*

Dear/s. Prof. Dr. Salwa Farouk Ali Sabet, Assoc. Prof. Dr. Sherif Abdel-Aziz Ibrahim, Assoc. Prof. Dr. Haidan Mostafa Elshorbagy and Mr. Amr Ahmed Abdelfattah.

This letter is to inform you that your following animal protocol / Project was approved by the CU- IACUC reviewers.

**Approval number:**

| | | | | |
|---|---|---|---|---|
| CU | I | F | 54 | 19 |

**Protocol Title:** EVALUATION OF THE POTENTIAL ANTICANCER EFFECT OF A NOVEL CHALCONE DERIVATIVE IN EHRLICH SOLID CARCINOMA-BEARING MICE

**Thesis / project title:** EVALUATION OF THE POTENTIAL ANTICANCER EFFECT OF A NOVEL CHALCONE DERIVATIVE IN EHRLICH SOLID CARCINOMA-BEARING MICE

First approval Date: Sept. 2019

Expiration Date: Sept. 2022

Please be advised that the CU-IACUC approval is limited to **one year** minimum. Any technical or administrative changes to the approved protocol must be submitted in writing to the CU-IACUC for approval. Changes should not be initiated until written CU-IACUC approval is received. Any adverse events should be reported to the CU-IACUC as they occur. When you need to extend this protocol the renewal must be submitted for approval at least three months prior to the expiration date of this approval.

***Best Regards***

**Prof. Khadiga Gaafar**
**Chair of CU - *IACUC***
