# Supplementary material for: [1,2,4] Triazolo [3,4-a]isoquinoline chalcone derivative exhibits anticancer activity via induction of oxidative stress, DNA damage, and apoptosis in Ehrlich solid carcinoma-bearing mice
Source: Naunyn Schmiedebergs Arch Pharmacol. 2022 Jul 26;395(10):1225–38. doi: 10.1007/s00210-022-02269-5 (PMC9467967; doi:10.1007/s00210-022-02269-5)
Supplement: Supplementary file 13 — Supplementary file13 (PDF 719 KB) [file 210_2022_2269_MOESM13_ESM.pdf]

Institutional Animal Care and Use Committee (CU-IACUC)
Cairo University

Cairo University

Dear **Dr. Amr WalyEldeen**

Upon your request, we confirm that Cairo University Institutional Animal Care and Use Committee (CU-IACUC) approved the euthanasia method performed (via Cervical dislocation under anesthesia) in the submitted protocol entitled **"EVALUATION OF THE POTENTIAL ANTICANCER EFFECT OF A NOVEL CHALCONE DERIVATIVE IN EHRLICH SOLID CARCINOMA-BEARING MICE"** which has been approved on September 2019 with approval number (**CU-I-F-54-19**) and expires in September 2022.

Best Regards

Prof. Khadiga Gaafar
Chair of CU - *IACUC*
